# Just how versatile are domains?

**DOI:** 10.1186/1471-2148-8-285

**Published:** 2008-10-14

**Authors:** January Weiner, Andrew D Moore, Erich Bornberg-Bauer

**Affiliations:** 1 Institute for Evolution and Biodiversity, Evolutionary Bioinformatics Group, Westphalian Wilhelms-University, Münster, Germany

## Abstract

**Background:**

Creating new protein domain arrangements is a frequent mechanism of evolutionary innovation. While some domains always form the same combinations, others form many different arrangements. This ability, which is often referred to as versatility or promiscuity of domains, its a random evolutionary model in which a domain's promiscuity is based on its relative frequency of domains.

**Results:**

We show that there is a clear relationship across genomes between the promiscuity of a given domain and its frequency. However, the strength of this relationship differs for different domains. We thus redefine domain promiscuity by defining a new index, *DV I *("domain versatility index"), which eliminates the effect of domain frequency. We explore links between a domain's versatility, when unlinked from abundance, and its biological properties.

**Conclusion:**

Our results indicate that domains occurring as single domain proteins and domains appearing frequently at protein termini have a higher *DV I*. This is consistent with previous observations that the evolution of domain re-arrangements is primarily driven by fusion of pre-existing arrangements and single domains as well as loss of domains at protein termini. Furthermore, we studied the link between domain age, defined as the first appearance of a domain in the species tree, and the *DV I*. Contrary to previous studies based on domain promiscuity, it seems as if the *DV I *is age independent. Finally, we find that contrary to previously reported findings, versatility is lower in Eukaryotes. In summary, our measure of domain versatility indicates that a random attachment process is sufficient to explain the observed distribution of domain arrangements and that several views on domain promiscuity need to be revised.

## Background

Domains are protein structural units that are also evolutionarily conserved on sequence level [1-3] (see also review by Bornberg-Bauer et al. [4] and references therein). While there are numerous combinations of domains (which we call *domain arrangements*), there are by orders of magnitude fewer domains.

Furthermore, a large part of most proteins can be mapped to known protein domains [5,6]. Thus, domains can be viewed as the building blocks of proteins: most known proteins are composed of a limited number of domains and some other structural units such as coiled-coils. Most domains are found in identical combinations in all proteins in which they occur. However, some domains form a large number of different combinations [7-9]. For example, the SH3 domain occurs in as many as 654 different arrangements in various proteins in the SwissPfam [10] database only.

The ability of a domain to form different combinations was first termed domain mobility [1,11] and this term is still sometimes used in the context of intron-wise modular recombination [12]. However, it turns out that novel combinations usually do not arise by domains being transferred from one protein to another, but by events such as the fusion of one protein with another or loss of terminal protein fragments [13-18]. Therefore, the term "domain versatility" or "domain promiscuity" seems to be more appropriate [4]. In the following, *promiscuity *will refer to a domain's tendency to combine with other domains to form different proteins. Understanding the tendencies of domains to form different combinations is crucial for understanding the evolution of multidomain proteins [19-24], genomic comparisons [20] and even findings of direct medical impact [25].

Several measures of domain promiscuity exist (see Tab. 1). One counts the number of different domains, with which the given domain occurs in a protein (*NCO*) [7]. However, with this approach, even domains that always co-occur in the same local context, ie. have the same neighbouring domains, have increased promiscuity. This is because there is no distinction between co-occurring domains that are positioned next to the domain of interest and co-occurring domains found at distant sites within a protein. A possible solution can be to count only the number of different immediate neighbours (*NN*) [26]. Tordai et al. [12] used yet another measure, in which they count in how many different triplets a domain occurs (*NTRP*). A unique triplet is composed of three domains – the domain of interest and its N- and C-terminal neighbours.

**Table 1:** Different measures used for asserting domain combination tendencies

Measure	Abbr.	Description	Reference
Co-occurrence	*NCO*	Number of domains that are found at least once in the same proteins as the given domain	[7]
Number of neighbours	*NN*	Number of direct neighbours found for a given domain	[26]
Number of triplets	*NTRP*	For a given domain *A*, number of different combinations *X – A – Y*, where *X *and *Y *are domains or N- or C-termini	[12]
Weighted bigram frequency index	π_*i*_	See original paper for exact definition	[32]
Domain versatility index	*DV I*	Strength of the relationship between the number of occurrences and the number of neighbours	this study

Domains and their ability to form different combinations can be represented as a graph, allowing the usage of network-analysis tools. A co-occurrence network can be constructed as follows: nodes represent domains and edges between two domains are drawn if both domains occur at least once in the same protein. Since most domains occur in only one combination, but few domains form a large number of combinations, the network is scale free [7,27]. Several investigations focused on such co-occurrence networks of proteins [7,20,21,25]. By analysing the co-occurrence graph, "hubs" can be identified. Such hubs correspond to domains that have a high number of links to other domains.

Few factors have been proposed to influence a domain's likelihood to form many combinations. First, a domain is less likely to form combinations if it does not have a robust, autonomous fold [28]. Secondly, Vogel et al. [29] have shown that the combination tendencies of domains can be explained by a preferential attachment model originally described by Koonin et al. [27]. In this model, domains that have a high abundance tend to form more combinations, i.e they are apparently more promiscuous. However, promiscuity is, in this model, a consequence of a random evolutionary process in which selection does not directly play a role. If this is the case, the concept of domain promiscuity (that is, that some domains have an inherent property that makes them more promiscuous than others) would be devaluated. For example, the fact that a domain is a hub in a domain co-occurrence network would not give more information than the statement that this very domain is widely spread. Another property of domains that seems to be connected with promiscuity is a domains average size in amino acids. Tordai et al. [12] have found a weak, negative correlation (*r *= -0.15) between domain size and *NTRP*, meaning that small domains tend to be more promiscuous. However, whether domain size is correlated with abundance remains to be tested. Furthermore, they have shown that certain classes of domains are more versatile than others. For example, extracellular proteins and proteins located on type 1-1 exons are found to form a higher number of triplets (*NTRP*).

Therefore, an important question arises: are all domains equally promiscuous given a certain number of occurrences? What observations concerning the causes of versatility are still supported if domain abundance is corrected for? Is the model of evolution completely random? In this study we show that, while taking a simple measure of domain promiscuity (such as the number of co-occurrences) is misleading, there is a property of domains that (i) is not simply correlated with the frequency of a domain, and (ii) corresponds to a domain's tendency to form combinations. We propose that a measure, which will be defined in the following, should be used that disentangles a domain's promiscuity from its frequency. We call this measure *domain versatility index*.

## Results and Discussion

### Measures of domain promiscuity and the domain versatility index

#### Existing measures of domain promiscuity

**Figure 1:**
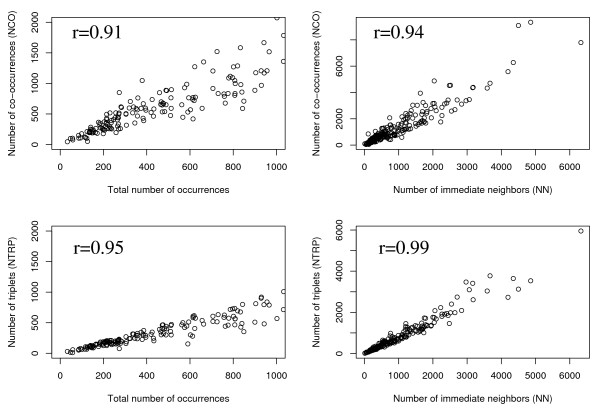
**Comparing different measures of domain promiscuity**. Comparison of the different measures of versatility showing that they are correlated with the number of occurrences of a domain. Data were obtained from Pfam (for details refer to methods). Each point represents a different domain. **Left**, correlation with the number of occurrences of a domain. **Right**, correlation with the number of immediate neighbours. *N *– number of occurrences, *NCO *– co-occurrences, *NN *– number of direct neighbours, *NTRP *– number of triplets. Spearman rank correlation coefficients between the different measures are given in the respective panels.

First we asked whether different measures for asserting domain promiscuity produce similar results. We compared the following methods: number of different direct neighbours of a given domain (*NN*), number of domains with which a given domain occurs in the same arrangement (*NCO*) and number of triplets (*NTRP*) (Tab. 1, see Methods for details). We found that all applied measures strongly correlate with each other and with the number of occurrences of a given domain (Fig. 1). Thus, most of the variation in domain promiscuity as defined by any of the existing measures can be explained by the number of occurrences.

The data presented further were obtained using the number of neighbours (*NN*) as a preliminary measurement for promiscuity. However, the results are similar if other measures are selected for computation (see supplementary material).

#### Correlation between number of occurrences (N) and number of observed neighbours (NN) across genomes

**Figure 2:**
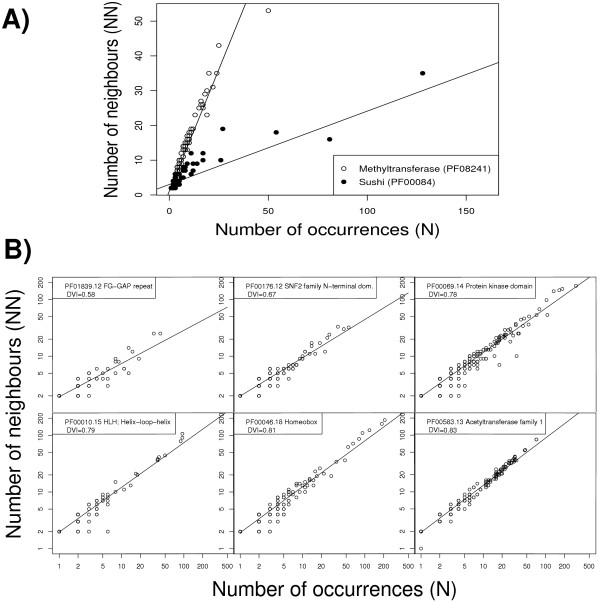
**The relationship between N and NN for selected examples**. **A**) Correlation between the number of occurrences (*N*) and number of neighbours (*NN*) for the methyltransferase domain (PF08241) and the Sushi domain (PF00084) (corrected for repeats, see Methods, DVI calculation). Each data point corresponds to the number of occurrences and the number of neighbours that a domain has in one genome. **B**) Correlation between the number of occurrences (*N*) and number of neighbours (*NN*) for selected domains. Each data point the corresponds to the number of occurrences and the number of neighbours that a domain has in one genome. Domain ID, description and *DV I *are given in the left upper corner of the respective graph. For a definition of *DV I*, see section "The domain versatility index".

To understand the relationship between the number of occurrences of a domain and its promiscuity, we investigated the following question in more detail. If the number of observed neighbours correlates with number of occurrences, does, for a given domain, the number of its neighbours *in a given genome *correlate with the number of occurrences of that domain in that genome? In other words, assume one counts the number of occurrences (*N*) of a certain domain in different genomes. For each genome, we compare *N *with the number of neighbours this domain has in the given genome. We wanted to know if there is a correlation, in a given genome, between the number of occurrences of a domain and the number of its neighbours. First, we divided the SwissPfam database in sets, each corresponding to one organism only. Secondly, we compiled a list of all domains found in SwissPfam. For each of these domains, we analysed each of the genome files separately. Given the domain and organism, we counted the number of occurrences (N) of this domain as well as the number of its neighbours (*NN*). For each domain, we analysed the relationship between *NN *and *N*. A comparison of this relationship for two domains and sample plots for selected domains are displayed in Fig. 2. Each of these plots corresponds to a domain or domain clan (see Methods for the description how calculations were made for domain super-families; domain clans were taken from Pfam [10]).

Many domains can be found in only one or two genomes, or are always present in one or two different arrangements only. We find that 86,252 Pfam A and B domains (74%) are found only in one or two proteins in the SwissPfam data set. 77,629 domains (66%) have only two or fewer different neighbors. For Pfam A domains the corresponding numbers are 1624 (26%) and 1265 (20%), respectively. Therefore, no regression can be calculated for these domains. However, for the domains for which a correlation could be reliably calculated, all of them show a significant correlation between *N *and *NN*. In fact, the relationship between *NN *and *N *is almost perfectly log-linear for many domains (see Fig. 2 and supplementary material). We find that, for a given domain, the number of occurrences in a genome is a very good predictor of promiscuity when promiscuity is defined by *NN*, *NCO *or *NTRP*. The tight relationship between *N *and *NN *underlines the fact that measures such as the number of co-occurrences, neighbours or local triplets of a protein essentially only show how abundant a domain is.

According to the previously proposed random model of evolution [29], the promiscuity of a domain defined in terms of co-occurrences is tightly linked to the frequency of a domain. If this was the case, then the apparent promiscuity of domains does not necessarily depend on any inherent property of the domain itself. Instead, it may indicate that such domains had more opportunity to propagate and rearrange, for example because they are older. In other words, given the high correlation between *N *and *NN*, domain promiscuity based on *NN *does not give substantially more information than its abundance. To understand how domains differ in their inherent properties to form new combinations, these two factors – number of abundance and promiscuity – must be unlinked. This prompted us to search for a new measure of promiscuity that would not be correlated with domain abundance. Using this measure, it should be possible to find out which properties of domains correlate with their abundance, and which show the intrinsic ability of a domain to form new combinations.

We observed that the strengh of the relationship between *N *and *NN *differs for different domains. Fig. 2A shows an example of two domains – a methyltransferase domain and the Sushi domain. For both domains, there is a strong relationship between the number of occurrences (N) and the number of neighbours (*NN*). However, given the same *N*, the methyltransferase domain has many more neighbours than the Sushi domain.

### The domain versatility index (DVI)

Although, as shown above, several measures of promiscuity are actually measures of their abundance, we see varying dependency of all these measures on the number of occurrences of the domain (e.g. of *NN *on *N*) for different domains. We argue that this measure reflects the concept of versatility better than other measures which are correlated with domain abundance.

**Figure 3:**
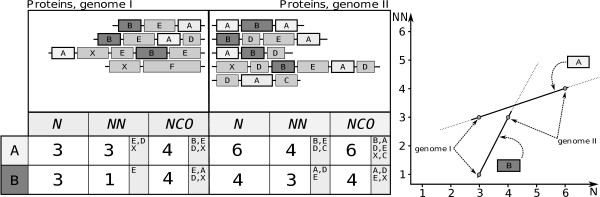
**Examplary calculation of the DVI**. Exemplary calculation of the *DV I*. Sets of proteins belonging to two distinct genomes are indicated as strings of domains represented by boxes in the top left. The occurrence of two exemplary domains, A and B, is displayed in the table, along with two measures of domain promiscuity. *N *denotes the total occurrence, *NN *the total number of direct neighbours and *NCO *the total number of co-occurrences for a given domain in its respective genome. Grey shaded fields within the *NN *and *NCO *fields indicate the specific domains that yield the respective values. In essence, the *DV I *represents the strength of the relationship between *N *and *NN*, indicated by the graph to the right. Each line represents a domain as indicated by associated boxes. The slope for the two domains, A and B, signifies the *DV I*. The desired unlinking of the versatility measurement from the total occurrence is clearly illustrated; despite the overall lower occurrence of domain B, it tends to form new combinations more readily indicated by the steeper slope in the relationship between *N *and *NN*.

Let us consider the following theoretical example. Let domain *A *have a large number of neighbours (Fig. 3). In the genome I, it occurs three times (*N *= 3) and forms four combinations in which it has altogether three different neighbours (*NN *= 3). In the same genome, another domain *B *has three occurrences but only one neighbour (*N *= 3, *NN *= 1). If we take *NN *as the measure of promiscuity, *B *will appear to be less promiscuous than *A*. Next, let us consider the situation in a second genome II (Fig. 3). Here, domain *A *has 6 occurrences and four neighbours, while domain *B *has 4 occurrences and 3 neighbours. Thus, the *NN *of domain *B *is still smaller than the *NN *of *A*. However, we see that, despite the fact that domain *B *occurs only once more, it gains two neighbours while domain *A *occurs three more times gaining only a single new neighbour. It seems as if domain *B *has some property that, illustrated in the relationship between *NN *and *N*, makes *B *more readily form new combinations despite it's overall lower frequency in comparison to domain *A*. We thus wanted to obtain a parameter that describes a domain's tendency to from combinations, but is independent of the frequency with which a domain occurs in a given genome. Therefore, we defined the *Domain Versatility Index *(*DV I*) as the strength of the relationship between the number of occurrences of a domain and the number of its neighbours. More precisely, we calculate the logarithmic regression of *NN *over *N*, and take the linear coefficient as *DV I*.

We observe that different domains exhibit different versatility. In essence, the DVI measures how likely a domain is to gain new combinations given a certain abundance in a certain genome. We measure the DVI by comparing abundances and domain combinations in different genomes. Each data point shows how this relationship evolved in a given context of genome evolution. Genomes evolve, among others, by genome and gene duplications during which the abundance of domains increases. How many new combinations are formed after such events through modular rearrangements depends on the ability of a domain to gain these new combinations. Thus, if we observe a strong linear relationship across genomes and even kingdoms, we conclude that there are intrinsic, domain-specific constraints that act on the evolution of domain combinations throughout the whole evolutionary history. It is therefore important to calculate the DVI of domains in many genomes to investigate how domain arrangements evolve.

Calculating the *DV I *can be done using different approaches. For example, one can include or exclude Pfam B domains. Furthermore, as only few data points exist for many domains, some of the *DV I *values are loaded with large regression error. Thus, it seems reasonable to select a cut-off for the percentage error. We only considered domains, for which the regression coefficient error was smaller than 10%.

Additionally, the number of neighbours is limited by the number of occurrences. In any given protein a domain can have at most two neighbours. Thus, if a domain occurs once, it can only have two neighbours; if it occurs twice, it can only have at most four neighbours. More abundant domains rarely achieve the maximum possible number of neighbours. However, domains occurring with a low copy number in a genome tend to approach the limit, thus facilitating a steeper slope of the regression line for small *N*. Thus, for computational purposes, it is reasonable to select only those domains for calculation that occur in at least one genome with at least a given number of neighbours. We tested several approaches. For example, we tested regression models more complex than log-linear, simple linear regression without logarithmic transformations and different versatility measures (*NTRP *or *NCO*) than the number of neighbours (*NN*). All the different calculation procedures give similar results, and the obtained coefficients are highly correlated (see supplementary material). The different *DV I *sets calculated using different approaches and the overall *DV I *distribution correlate (Pearson *r *correlation coefficient > 0.8).

Furthermore, our initial analyses have shown that domains that form repeats (for example, LRR 1 or Ankyrin repeat) have an unusually low *DV I *(see supplementary material). One can explain this by the fact that most occurrences of these domains fall within a repeat stretch, while the expansion of protein repeats [30] does not increase the actual number of neighbours. To remove this effect, we considered stretches of one repeated domain as a single occurrence following the approach by Ekman et al. [31]. Here, we present results of the DVI calculation using restrictive thresholds. We only calculated the *DV I *for Pfam domains that (i) occur in at least 3 genomes, (ii) have at least 20 neighbours in at least one genome and (iii) have a low error (error of the *DV I *< 10%). In this set, we were able to define a *DV I *for 358 Pfam A domains and 58 Pfam B domains. The distribution of the calculated *DV I *is roughly normal with mean 0.60 ± 0.14. If the constraints are relaxed, the results are similar; however, the error of the calculated coefficients is larger.

Furthermore, as expected, the *DV I *does not correlate either with the number of occurrences, *N*, or with other promiscuity measures such as number of neighbours, *NN *(Fig 2, supplementary material). The full results tables can be downloaded from the supplementary material web page.

We find low *DV I *values for several repeat domains such as the extensin domain (Extensin_1) or leucine rich repeat (LRRNT), despite having corrected for the number of repeats. Also, other domains such as Sushi or the Receptor L domain which are very frequent have a low *DV I*. Domains with a high *DV I *include several transcription related genes such as Zinc finger, bZIP or basic helix-loop-helix domain (bHLH), but also the caspase recruitment domain (CARD), chaperone domain DnaJ and different transferase domains (Tab. 2). In order to be able to calculate the *DV I *for a given domain, the domain in question must occur in several genomes. Thus, the *DV I *cannot be calculated for domains that are not widely spread, for example because they occur only in a few known proteins. The problem of the low coverage of the PFAM database by domains with a calculated DVI has, in fact, two solutions. Obviously, with each new genome sequenced and annotated, the amount of data for evaluation will grow and thus also the number of domains that have an assigned DVI. In fact, we used the complete proteome data from Integr8 database [50] to analyse the DVI of 749 full genomes. This increased the number of Pfam A domains with a DVI assigned to 727, and the data obtained was almost identical to the *DV I *derived from the SwissPfam database (*r *= 0.9). Furthermore, it turns out that the ratio $\frac{NN}{N}$ is significantly correlated with DVI (*r *= 0.7, *p *< 10^-15^; see supplementary data) and thus can be used as a proxy.

**Table 2:** Domain Versatility Indices (*DV I*) for 30 selected Pfam A domains. *DV I*, the domain versatility index. Err, the calculated error of the regression coefficient. Description, description as taken from the Pfam database.

			Domains with a low *DV I*
Domain	DVI	± *SE*	Description
PF02861.9	0.231	0.015	Clp_N; Clp amino terminal domain
PF06815.2	0.236	0.014	RVT_connect; Reverse transcriptase connection domain
PF00353.9	0.244	0.022	HemolysinCabind; Hemolysin-type calcium-binding repeat (2 copies)
PF00030.8	0.269	0.021	Crystall; Beta/Gamma crystallin
PF03130.5	0.275	0.020	HEAT_PBS; PBS lyase HEAT-like repeat
PF00009.15	0.276	0.006	GTP_EFTU; Elongation factor Tu GTP binding domain
PF00402.7	0.282	0.020	Calponin; Calponin family repeat
PF00954.11	0.288	0.028	S_locus glycop; S-locus glycoprotein family
PF00228.9	0.296	0.018	Bowman-Birk_leg; Bowman-Birk serine protease inhibitor family
PF02012.9	0.306	0.030	BNR; BNR/Asp-box repeat
			
			Domains with a medium *DV I*
Domain	DVI	± *SE*	Description

PF02362.12	0.626	0.014	B3; B3 DNA binding domain
PF01825.10	0.627	0.035	GPS; Latrophilin/CL-1-like GPS domain
PF07721.4	0.627	0.025	TPR_4; Tetratricopeptide repeat
PF01302.13	0.628	0.031	CAP_GLY; CAP-Gly domain
PF00176.12	0.630	0.009	SNF2_N; SNF2 family N-terminal domain
PF00567.14	0.631	0.039	TUDOR; Tudor domain
PF01390.8	0.631	0.044	SEA; SEA domain
PF07686.5	0.632	0.016	V-set; Immunoglobulin V-set domain
PF00067.11	0.633	0.012	p450; Cytochrome P450
PF00165.11	0.633	0.009	HTH_AraC; Bacterial regulatory helix-turn-helix proteins, AraC family
PF00249.19	0.635	0.014	Myb_DNA-binding; Myb-like DNA-binding domain
			
			Domains with a high *DV I*
Domain	DVI	± *SE*	Description

PF00004.18	0.826	0.006	AAA; ATPase family associated with various cellular activities (AAA)
PF00583.13	0.827	0.006	Acetyltransf_1; Acetyltransferase (GNAT) family
PF00250.7	0.828	0.011	Fork_head; Fork head domain
PF01926.11	0.828	0.011	MMR_HSR1; GTPase of unknown function
PF00001.11	0.830	0.011	7tm_1; 7 transmembrane receptor (rhodopsin family)
PF01464.9	0.846	0.025	SLT; Transglycosylase SLT domain
PF04055.9	0.857	0.006	Radical_SAM; Radical SAM superfamily
PF00496.11	0.858	0.015	SBP_bac_5; Bacterial extracellular solute-binding proteins, family 5 Middle
PF02393.6	0.872	0.040	US22; US22 like
PF08241.1	0.911	0.007	Methyltransf_11; Methyltransferase domain

Finally, the low number of the DVIs obtained was partly due to the rigorous thresholds applied. Relaxing the thresholds we obtain a DVI for over 1,200 Pfam A domains. We have repeated the analyses with this data set and found that our findings remain unchanged (see supplementary material). Thus, the relatively small coverage of Pfam A+B is not an inherent limitation, because it depends only on the amount of available sequence information. With the growing number of completely sequenced genomes, it will become possible to calculate this property for many more domains.

A recently published method by Basu et al. [32] tries to adress the problem of domain versatility in a similar manner. The method also builds upon a calculation of normalising the number of neighbours of a given domain by its frequency. However, our method corrects for gene expansions within a genome and for domain expansions; furthermore, since it includes both PfamA and PfamB domains, a possible problem with low coverage of amino acid sequences is avoided. Last but not least, the DVI corresponds directly to the evolutionary expansion of domain neighbourhood as a result of the increase of domain abundance (Fig. 2). Thus it reflects directly an evolutionary process and can be easily interpreted as the likelihood of a domain to form new combinations.

To compare DVI with the weighted bigram frequency index presented by Basu et. al [32], we applied the goodness of fit statistics *R*^2 ^to the DVI (linear and logarithmic regression) of every domain and to a regression model that corresponds to the weighted bigram frequency index. We find that the logarithmic regression model outperforms the bigram frequency index in 95% of the cases and the linear model in 62% of the cases. The average difference between the DVI (defined by the logarithmic regression model) and the weighted bigram frequency index is 12%.

### What makes a domain versatile?

The hypothesis that some domains might be more versatile than others – irrespectively of whether they are more frequent – is intriguing. We wanted to know whether, given the same frequency of occurrence, some domains will propagate and form new combinations more easily than others. We attempted to elucidate whether a certain property of a domain influences its versatility. Therefore, we explored the following possible links to the *DV I*: domain age, length and pattern in forming domain arrangements. Furthermore, we tested the influence of genetic mechanisms on the *DV I*. In particular, we studied the influence of LTR retrotransposons on the *DV I *because of the clear diagnostic features they exhibit and the established methods available to detect them. Retrotransposons can occasionally move non-viral domains and could thus enhance domain recombination rates. We also wanted to know whether domains with a certain function have a higher *DV I*. It is conceivable that, depending on a function and particular selective pressures, some domains may be more "useful" for the organisms in different protein contexts than others. For example, domains that are responsible for protein-protein interactions or DNA binding should be more likely to occur in different combinations. Vogel et al. [29] explored the relationship between domain abundance and number of neighbours (*NN*), however they did not find any conclusive results.

#### Versatility and domain age

We examined whether domains that originated early in life history have a higher number of neighbours, as predicted by the random evolution model. To get a rough estimate of the relationship between the age of a domain and its properties, we have adopted a simplified version of the approach described by Wang et. al [33] and divided domains into three groups: "old", "middle" and "new" based on their taxonomic spread. "Old" domains are present in all kingdoms; "middle" are common for all lineages in either of the Eukaryota, Archea or Bacteria and "new" are specific to one lineage within these groups (e.g. in Metazoa only).

For example, domain PF00001 (a transmembrane receptor from the rhodopsin family) is found in all domains of life, while the Kringle domain (PF00051) can be found only in the eukaryotes. While the former would be defined as 'old,' the latter would be defined as 'new' and nodes in-between as 'middle.' Indeed, domains that originated early have, on average, a higher number of neighbours. For example, domains that are found in all main three life forms (Bacteria, Archea and Eukaryota) have on average 12.51 neighbours, while the respective values for Eukaryota and Metazoa are 5.72 and 3.69. This does not mean that all ancient domains are very widely spread or have a high number of neighbours. In fact, the variance of these properties also increases with domain age, and thus there are domains that probably are very ancient, but do not form many combinations. For example, the RNA polymerase domain PF04983 occurs in many clades altogether 89 times, but has only 9 different neighbours.

However, we expect that truly versatile domains should have equal chance of arising during the course evolution. That is, a young domain that recently appeared can also be versatile, albeit in a limited clade. For example, a novel transcription related domain that recently evolved in mammals is not expected to be different in its ability to form new domain combinations from a domain shared throughout the tree of life, although it will not have yet as many connections formed. Given the random model, the number of connections is a function of time, however not necessarily dependent on an inherent property different in older and in younger domains. We observe a strong correlation between the age of a domain and its observed number of occurrences, as well as the number of neighbours (*p *< 10^-15 ^in a one-way ANOVA for three age categories). Therefore, older domains seem to be more promiscuous if measured using previously described units of promiscuity such as the *NN *or *NCO*. This is possibly due to the fact that these domains had more time to spread, in accordance with the model presented by Vogel et al. [29].

**Figure 4:**
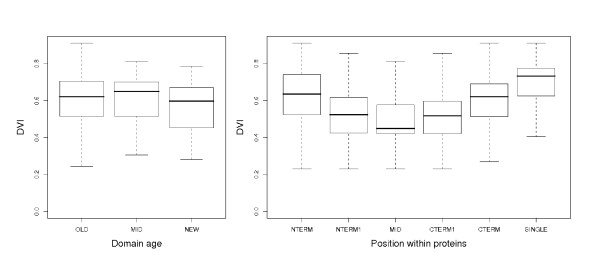
**The relationship between the DVI, domain position and domain age**. **Left: **Domain age and the *DV I*. OLD – domains that are common to all three main branches of life (Bacteria, Archea, Eukaryota); MID – domains that are present in all taxons of one of these branches (e.g. domains that can be found only in Bacteria, but not in Archea or Eukaryota); NEW – domains that are present only in one subgroup of one of these branches (e.g. domains that occur only in vertebrates). **Right: ***DV I *and position of the domain within the protein. NTERM – N-terminal domains; NTERM1 – next-to N-terminal domains in proteins with four domains or more; CTERM – C-terminal domains; CTERM1 – next-to N-terminal domains in proteins with four domains or more; MID – all remaining (non-terminal) domains; SINGLE – domains in single-domain proteins. On the *y *axis, domain versatility index (*DV I*). Bold line denotes the median; boxes denote the firstand second quartiles; whiskers show the minimum and maximum values not including outliers.

We wanted, however, to know, whether older domains are intrinsically more versatile than younger domains. Specifically, we ask whether domains that originated at the root of the tree of life have a significantly different *DV I *from domains that originated later. We did not find any significant differences (*p *= 0.16 in a one-way ANOVA). Since the domains that are specific to a clade are generally younger than domains spread throughout the tree of life, this finding shows that the average *DV I *does not depend on the age of a domain (Fig. 4). This means that domains arising at any time during the evolution have the same chance of becoming versatile.

We further asked whether the domain versatility differed significantly between Bacteria, Eukaryotes and Archea. We expected that due to the fact that eukaryotic proteins tend to contain more domains, the versatility in eukaryotic proteins will be higher. To test this hypothesis, for each of the genomes in one of the three forms of life (Bacteria, Archea and Eukaryota) we calculated the average *DV I *of all domains that are contained in that genome.

We find a small, but statistically highly significant difference between prokaryotic (bacterial and archeal) and eukaryotic genomes. The average *DV I *for bacterial, archeal and eukaryotic genomes were, respectively, 0.64 ± 0.0003, 0.65 ± 0.0012, and 0.58 ± 0.0002. Thus eukaryotic domains tend to be even slightly *less *versatile, although though they may form, on average, more connections in a domain co-occurrence network than a prokaryotic domain. This is in contrast with previously reported findings based on domain versatility measures correlated with domain abundance [12]. There are two possible explanations for this phenomenon. First, it may be that the apparent decrease in DVI corresponds to a higher rate of gene expansion by gene and genome duplications. We have tested this by correcting the DVI by gene expansion through removal of redundant domain arrangements and found that results are robust. A second possibility is based on the fact that eukaryotic proteins have, on average, more domains than prokaryotic protein. Furthermore, it has been previously reported that domain rearrangements most frequently involve protein termini [18]. Since in Eukaryota, there are proportionally fewer terminal domains and since the non-terminal domains are less likely to acquire new neighbors, on the average, an eukaryotic domain will form fewer connections (acquire new neighbors) than expected from its abundance, and therefore has a relatively low *DV I*. On the same hand, due to the larger genomes and gene expansion in Eukaryotes, domains are more abundant and domain rearrangements more frequent in terms of absolute numbers. This makes the domains appear more versatile if one applies one of the measures of promiscuity that is correlated with domain abundance.

#### Correlations between DVI and arrangements properties of domains

It has been shown that domain rearrangement events very often involve whole terminal fragments [17,34,35]. Such rearrangement events, which are predominantly protein fusion and fission events [16,36], very often involve single domain proteins that are fused to or split from another protein. We observed that domains which also occur as single-domain proteins have, on average, a higher *DV I *(0.62 ± 0.01 vs 0.53 ± 0.01; *p *< 10^-7 ^in a two-tailed t-test with equal variances). We further supported this finding as follows. For a given domain, we considered all proteins in SwissPfam that contain this domain, and calculated the fraction of single-domain proteins (that is, fraction of proteins in SwissProt that harbour only this given domain). The proportion of single-domain proteins is correlated with the *DV I *of the domains (Pearson *r *= 0.25, *p *< 10^-5^). Therefore, if a domain occurs frequently as a single domain protein, it is likely to have a high *DV I*.

Some domains may be more prone to be rearranged by a specific genomic mechanism. For example, retrotransposons are abundant in many eukaryotic genomes and may play a condsiderable role in their evolution [37-40]. It has be suggested that retrotransposons may promote recombination [37,41,42] and thus may also have an impact on domain rearrangements in turn facilitating a higher *DV I*. To test whether domains that are included in retrotransposition events tend to have a higher *DV I*, we used the LTR detection method described by Rho et al. [43]. We scanned genomic DNA from *Mus musculus *for long terminal repeats, translated the nucleic acid sequence flanked by LTRs into all six reading frames and detected Pfam A domains by running HMMPFAM from the HMMER package [44] against Pfam A HMM models. Using an E-value cut-off of 10^-5 ^and removing common viral domains such as Transposases, RVTs or Gag-related domains, ~180 mostly non-viral domains were obtained. We were able to calculate the *DV I *for 53 domains, but did not detect any significant difference between domains found within LTR boundaries and other 'non-LTR' domains.

Previously, we have shown that domains in terminal indels differ with respect to their number of neighbours from domains found on non-terminal positions. We now asked, whether domains found at the termini are more often partaking in fusion/fission events simply because they are more abundant, or whether they tend to be more versatile. We found that the latter is the case. The average *DV I *for terminal domains is 0.61 as opposed to 0.49 for the non-terminal domains (*p *< 10^-15 ^for a one-way ANOVA). This means that, on average, terminal domains are more versatile.

#### DVI and intrinsic domain properties

Furthermore, the *DV I *correlates with average length of a domain: longer domains have a higher *DV I *(*r *= 0.25, *p *< 10^-6^). This is in variance with previous investigations, which were based on other versatility measures such as the number of triplets (*NTRP*) formed [12]. We interpret this contradiction as follows. Number of triplets correlates strongly with domain abundance (*N*). It is therefore possible that domain length primarily correlates with domain abundance. Indeed, we see a statistically significant, negative correlation between the number of occurrences of a domain and the domain length (*r *= -0.14, *p *= 0.005). We also explored the link between domain structure and versatility, but did not find any correlation.

#### DVI and domain function

Next, we asked what the functional properties of domains with different DVI values are. A manual investigation of the domain functions has shown that domains with low *DV I *are very often repeats. Note that the combinatorial effect of the repeats (that is, that several occurrences of a domain in a row give a high number of occurrences and a very low number of neighbours) have been accounted for as described in the previous section.

Among the domains with the highest *DV I *(see supplementary table 1) there are several domains that bind to various nucleotides such as DNA, RNA and ATP binding domains. For example, we find several Zinc finger domains, the PAS fold or the Helicase domain. On the other hand, among the domains with low *DV I *we see numerous structural domains and domain repeats, such as the cap region of the leucine rich repeat (LRRNT), collagen triple helix (Collagen) or the Ankyrin repeat (ANK). We wanted to know whether these observations can be quantified and if they are statistically significant.

To quantitatively test the differences, we analysed the GO terms that are associated with different domains. A GO term was associated with a domain, if it was shared by all proteins containing the given domain, as defined in the Interpro database [45]. We calculated the average *DV I *for different GO terms and compared them using analysis of variance (ANOVA). We were not able to find any significant differences in average *DV I *between the different GO terms.

Since the calculation of a *DV I *is limited to domains that occur in multiple instances in several genomes, only a small fraction of Pfam A domains could be associated with a *DV I*. To alleviate this problem we calculated the *DV I *for the 263 Pfam clans. For example, instead of calculating the *DV I *separately for SH3_1, SH3_2, SH3_3, SH3_4 and SH3_5 domains, we treated each occurrence of such a domain as the occurrence of its respective clan ID "CL0010". In summary, we were able to calculate the *DV I *for 115 clans. Furthermore, we calculated the average *DV I *of Pfam domains that belong to a given Pfam clans and found that both measures are significantly correlated (*r *= 0.6, *p *< 10^-12^). While the latter (averaging *DV I *over members of Pfam clan) seems to be a more straightforward approach, the former (substituting domain names in SwissPfam by the respective Pfam clan IDs) allows a calculation of the *DV I *for a larger number of Pfam clans. We see that, for most of the Pfam clans, the correlation between *N *and *NN *is equally strong as for Pfam domains (see supplementary material).

We further repeated the GO analysis for Pfam clans. Again, we do not find a clear connection between the GO functions associated with a Pfam clan and the calculated *DV I*.

While we could see that protein domains from the same Pfam clan do indeed have similar *DV I *values, we were not able to show that domains that have either a low or a high *DV I *have a particular functional assignment, i.e. common GO annotations. Potentially, there could be a problem with the way GO annotations are assigned to proteins and protein domains; for example, automatically assigning GO terms to a domain by taking over the GO terms of the proteins that share the given domain may introduce a systematic error. On the other hand, there is maybe no general functional link between protein domain versatility and its function (e.g. some transcription factors are specialised and non-versatile whereas some others are versatile).

## Conclusion

The idea that some domains can more easily form new domain combinations than others is very intriguing. It is consistent with a model of protein evolution in which proteins with new functionality arise by combining functional units. A frequently used metaphor is the one of Lego blocks: complex proteins can be created by combining existing blocks of a simpler function. Just like the idea that new genes can be invented by neo- or subfunctionalisation [46,47], this perspective could shift the view we have on protein evolution. The arising of complex features can often be explained more easily from the perspective of modular evolution than using the classical models of molecular evolution such as evolution by point mutations. It is therefore not surprising that several investigations emphasize the concept of domain versatility or promiscuity.

We show that the previously used measures of promiscuity are coupled to the frequency of a domain in a genome. Therefore, to understand the principles of domain-wise modular evolution, one needs to disentangle these concepts and decouple the promiscuity of a domain from the frequency with which it occurs.

We have found a new measure, the domain versatility index (*DV I*), for what can be truly considered the versatility of a domain and have shown that this measure does not correlate with domain abundance or age. We have shown that this measure has properties expected from a measure of domain versatility. We find that domains that form single domain proteins or that occur at protein termini (where rearrangements occur frequently) have all a higher than average *DV I*. We also show that domain versatility, as expected for an intrinsic property, is not related to domain age. Furthermore, contrary to findings based on abundance-related versatility measures, we could not observe that eukaryotic domains are significantly more versatile. Also, we could not correlate the versatility with protein function or retrotransposition. We suggest follow-up studies should further consider the issue of linking DVI to the phylogenetic history of proteins, explaining when the proteins acquire new domain combinations and how the new combinations formed relate to the *DV I*.

## Methods

### Databases used and dataset preparation

We used SwissPfam from the Pfam database version 21 as of 2006 [10]. The SwissPfam set of protein arrangements was divided into files, each corresponding to one organism. In total, 10,746 species were included in the analysis. The files were converted to XDOM [48] format and can be downloaded from our web site. Interpro version 14 [45] was used to obtain GO annotations of Pfam domains. The mouse genome (NCBI m36) was obtained from the Ensembl [49] website. All supplementary material is available online at: http://iebservices.uni-muenster.de/suppmat/DVI/.

### Calculation of versatility parameters

#### Calculation of the direct indices of domain versatility

We calculated the following previously described indices of domain versatility: *number of co-occurrences *(*NCO*; [29]), *number of direct neighbours *(*NN*) and *number of triplets *(*NTRP*; [12]). For each domain, we calculated the number of co-occurrences as follows. For a given domain, all proteins containing this domain were identified. *NCO *is the total number of different domains in these proteins minus one. Number of immediate neighbours was calculated as the number of different domains that occur directly at the N- or C-terminus of the given domain. If a domain was found at a terminus, the number of neighbors was increased by one (terminal position of a domain was considered to be a valid domain combination). Triplets are defined as unique combinations of either: three domains, with the given domain in the middle, two domains and a letter if the domain occurs at either N- or C-terminus (e.g. N-PF00001–PF00002 means that the given domain PF00001 occurs at the N-terminus of the protein) or three letters, if the domain occurs in a single domain protein (e.g. N-PF00001-C). We calculated the number of triplets as the number of unique triplet combination in which a domain occurs. These calculations were done for all domains in the data set.

#### DVI calculation

We calculated the domain versatility index (*DV I*) as follows. For each domain, we considered the number of occurrences (N) of this domain in a genome as well as the number of immediate neighbours (*NN*). We define *DV I *as the coefficient of logarithmic regression of *NN *over *N *with its respective error.

Furthermore, we calculated and compared both, logarithmic and linear regression coefficient of *NCO*, *NN *and *NTRP *over *N*. We have chosen the logarithmic regression coefficient for further analysis, because it follows the logarithmic model of Vogel et al. [29]. The *DV I *has been computed in R using the following models: *NN *= *a *log(*N*) + *b *for logarythmic regression and *NN *= *aN *+ *b *for linear regresion (*N *– number of occurences, *NN *– number of neighbors, *a *and *b *– regression coefficients). The *DV I *was then defined as the *a *coefficient from the regression model.

To correct for domain expansion, we have removed domain repeats from the data set. Similarily, to correct for gene expansion, we have removed identical domain arrangements from the data set.

To test whether the use of SwissPfam introduces a significant bias to the observed data, we have recalculated the DVI using proteins from 749 full Eukaryotic, Bacterial and Archeal genomes from the Integr8 database, release 84 ([50]; see supplementary materials for the full data set). Overlapping domains were removed, with PFAM domains given precedence over other annotations. In this data set we obtained DVIs for 980 domains, including 727 Pfam A domains. These DVI values correlate significantly with the values obtained for the SwissPfam data set (*r *= 0.91, *p *< 10^-15^).

To compare different approaches for the DVI calculation and the index described in [32], we used the *R*^2 ^statistic to evaluate the goodness of fit for each domain and for different models applied. The equation used by Basu et al. [32] corresponds to a model given by the equation *NN *log(*NN*) = log(*N*); consequently, we define *NN' *= *NN *log(*NN*) and use the following regression model in R: *NN' *= *a *log(*N*) + *b*. We have calculated the average *R*^2^value for each of the three models applied.

### DVI for domains of different age

We defined three classes of domains. *"OLD" *– domains that are common to all three main branches of life (Bacteria, Archea, Eukaryota); *"MID" *– domains that are present in all taxons of one of these branches (e.g. domains that can be found only in Bacteria, but not in Archea or Eukaryota); *"NEW" *– domains that are present only in one subgroup of one of these branches (e.g. domains that occur only in Metazoa). The parameters (*DV I*, *N*, *NCO*, *NN*, *NTRP*) were calculated for these three groups and the significance of differences analysed with a one-way ANOVA.

### Analysis of Pfam CLANs

We calculated the *DV I *for Pfam clans using two methods. First, we calculated it as the average *DV I *of all the Pfam domains belonging to a clan for which a *DV I *could be obtained. Next, we modified the XDOM files by replacing each domain by its corresponding clan ID, and calculated the *DV I *as previously described for Pfam domains. Domains that do not have a CLANs assignment were retained.

### Statistical analysis

For statistical analysis, the R package [51] in the version 2.4.1 was used. For correlations, Pearson *r *correlation coefficient and Spearman rank correlation coefficient were used where appropriate. Principal component analysis of the different measures of versatility was done using the princomp method from the R package.

## Authors' contributions

JW designed the study, carried out the calculations and analysed the data. EBB supervised the study, evaluated the results and proposed additional experiments. RAM studied the influence of LTR retrotransposons, of the domain age and of the protein structure on the DVI. JW, EBB and RAM wrote the manuscript.
